# Microencapsulated Escape Lysine with Tannin as an Adjuvant in Sheep Diets

**DOI:** 10.3390/vetsci12010014

**Published:** 2025-01-01

**Authors:** Roberto Matheus Oliveira, José Morais Pereira Filho, Claudiney Inô, Évyla Andrade, Kevily Henrique Lucena, Juliana Paula Oliveira, Elzania Pereira, Ronaldo Oliveira, Ricardo Edvan, Leilson Bezerra

**Affiliations:** 1 Graduate Program in Animal Science and Health, Federal University of Campina Grande, Patos 58708110, Brazil; r.matheustavares98@gmail.com (R.M.O.); claudiney.felipe@estudante.ufcg.edu.br (C.I.); evylalayssa@hotmail.com (É.A.); hkevily@gmail.com (K.H.L.); 2 Campus do Sertão, Federal University of Sergipe, Nova Esperança, Nossa Senhora da Glória 49680000, Brazil; jupaula.oliv@academico.ufs.br; 3 Animal Science Department, Federal University of Ceará, Fortaleza 60455760, Brazil; elzania@hotmail.com; 4 Animal Science Department, Federal University of Bahia, Salvador 40170155, Brazil; ronaldooliveira@ufba.br; 5 Animal Science Department, Federal University of Piauí, Teresina 64049550, Brazil; edvan@ufpi.edu.br

**Keywords:** amino acid bypass, encapsulation, feeding behavior, protein, tannic, thermoregulate

## Abstract

The use of escape protein, which is absorbed in the small intestine, can improve the production of ruminant animals because it meets their protein requirements better. This study hypothesized that wax lipid matrices are effective encapsulants for escape lysine in ruminants and tested tannin extract as an adjuvant. Forty intact male Santa Ines × Dorper sheep (~4 months old, BW 23 ± 1.2 kg) were allocated, in a randomized block design, to four treatments: control group (no lysine addition), supplementation with free lysine, escape lysine, and escape lysine + tannin extract. The ether extract intake was higher in the sheep fed escape lysine + tannin compared to the control and free lysine group. The sheep fed escape lysine presented higher DM and aNDF eating efficiency (*p* ≤ 0.05) compared to the free lysine and control groups. Escape lysine with and without tannin presented greater TDN compared to free lysine and control. The NFCs were lower in the free lysine diet, and the crude protein digestibility was higher in the sheep fed lysine + tannin and the control group compared to the escape and free lysine groups. The lysine treatments did not affect (*p* > 0.05) the performance, ingestive behavior, and blood parameters. The rectal temperature was higher in the sheep fed lysine escape compared to those fed free lysine. The sheep fed escape lysine + tannin exhibited reduced (*p* ≤ 0.05) large particles and improved peNDF 12 h after feeding. The escape lysine diet, regardless of the addition of tannin, improved the total digestible nutrients and eating efficiency, without affecting the performance, feed conversion, and blood variables of the sheep.

## Simple Summary

Incorporating escape proteins into the diets of ruminants is essential for delivering key amino acids, such as lysine and methionine, directly to the intestine without being broken down in the rumen. Thus, this enhances protein utilization, improving growth, boosting milk production and overall animal health, and contributing to more efficient and sustainable livestock practices. This study investigated the effects of escape lysine protected in a lipidic matrix of carnauba wax associated with tannin extract from *Mimosa tenuiflora* on the ingestive behavior, selectivity, blood, physiological, and thermoregulation variables of sheep. Forty intact male Santa Ines × Dorper sheep were allocated into four treatment groups comparing the use of free and microencapsulated lysine in the diet. The results demonstrate that supplementation with escape lysine microencapsulated into a lipid matrix of carnauba wax, with or without tannin extract, positively influences the nutrient digestibility and eating efficiency, especially compared to the use of free lysine in the diet. This supplementation of escape lysine at a dose of 1.34% of the total dietary DM does not affect the sheep’s ingestive behavior, blood enzymatic activities, physiological parameters, or productive performance. Further research is needed to determine the optimal dietary inclusion levels, evaluate costs, and assess the impact of escape lysine on the meat quality of sheep.

## 1. Introduction

In ruminants, the quality of protein absorbed at the intestinal level is directly influenced by the types of amino acids included in the diet, the methods of dietary inclusion, and the way these amino acids are fermented by rumen microorganisms, especially in the formation of microbial protein. Essential amino acids like lysine may not reach the small intestine in sufficient quantities to meet the metabolizable protein requirements, particularly in high-yield animals [1,2].

When amino acids in their free form reach the rumen, they are rapidly degraded by microorganisms, leading to microbial protein synthesis. Consequently, the direct passage of amino acids to the small intestine is minimal, which is particularly concerning in high-production animals, where fermentation heat is more pronounced [1,3]. Using protected amino acids that can escape ruminal degradation is crucial, as they increase amino acid delivery to the intestine and prevent their catabolism by rumen microorganisms [2,4].

Several methods and materials have been tested to protect essential amino acids from microbial degradation, including formaldehyde [5], lipid-based systems (such as liposomes and solid lipid nanoparticles) [6,7], polymer-based systems (such as polylactic acid and polylactic-co-glycolic acid) [8,9], hydrogels [9], and natural proteins (such as zein and casein) [10]. Among lipid sources, waxes (such as carnauba and beeswax) have been highly recommended as coating materials to form a protective shell around amino acids [11,12]. This is largely due to the composition of carnauba wax (CW), which consists of fatty acid esters (80–85%), fatty alcohols (10–16%), acids (3–6%), and hydrocarbons (1–3%) [13]. Additionally, its high content of saturated fatty acids (SFAs) with long-chain esters ensures compatibility with ruminal microflora, as it does not degrade within this environment [14].

Medeiros et al. [11] emphasized that carnauba wax, known for its high hydrophobicity and resistance to digestion, can be an effective material. It is a natural wax with low degradability, and its wide availability makes it particularly suitable for controlled amino acid release [4,15].

Microencapsulation techniques aim to protect amino acid cores from physical, chemical, or biological degradation, ensuring their release at the desired site. The key to this process is achieving targeted release, improving the efficiency of dietary protein utilization, and maximizing the benefits of microencapsulation [4,16]. Additionally, tannins are recognized for their ability to protect nutrients from ruminal degradation. When incorporated at appropriate levels into ruminant diets, tannins can enhance nutrient digestibility and benefit the environment [17,18]. For instance, *Mimosa tenuiflora*, a legume native to the Caatinga biome, is rich in tannins and has potential for inclusion in ruminant diets [19,20,21].

The supplementation of escape lysine encapsulated with carnauba wax enriched with tannins could contribute to improving confined sheep production [16]. Such supplementation may enhance thermoregulation without negatively affecting serum parameters, physiological responses, ingestive behavior, or feed selectivity [22,23,24]. Furthermore, amino acids like lysine and methionine play a crucial role in thermoregulation by supporting metabolic processes essential for heat production and bodily maintenance. In heat-stressed environments, they optimize protein metabolism, reducing the thermal load on animals and improving heat dissipation through vasodilation. Therefore, this study tested the hypothesis that microencapsulated escape lysine, enriched with tannins, can improve production and thermoregulation in sheep, supporting their welfare and productivity under confinement.

## 2. Materials and Methods

### 2.1. Animals, Experimental Design, and Facilities

Forty intact male sheep, Santa Ines × Dorper crossbreeds, approximately four months old with an average initial body weight of 23 ± 1.2 kg, were used in this study. The animals were allocated, in a randomized block design, to four treatments and ten replicates per treatment. The treatments consisted of a control group without escape lysine inclusion (0.0%); a group with free lysine inclusion in the diet (non-encapsulated/0.44%); a group with lysine microencapsulated in a carnauba wax lipid matrix (1.34%); and a group with lysine microencapsulated in a carnauba wax lipid matrix combined with tannin extract from *Mimosa tenuiflora* (1.34%). The lysine inclusion levels were based on the NRC [1] recommendations for escape protein requirements for sheep.

At the start of the experimental period, all animals were weighed, identified, vaccinated against clostridial diseases and rabies, dewormed, and housed individually in suspended wooden pens (0.5 m above the ground) measuring 1.3 × 1.5 m^2^. The pens were equipped with feed and water troughs and located within an open-sided barn. The barn had a roof made of asbestos cement tiles, a central concrete floor, and slatted flooring, measuring 16 m in length and 6 m in width, with a 1.8 m wide central aisle and a ceiling height of 2.5 m. The experimental period lasted 70 days, including 15 days for animal adaptation to the environment, handling, and diets, followed by 55 days for sampling and data collection.

### 2.2. Preparation of Microencapsulated Systems, Ingredients, and Diets

Tannin was extracted from *Mimosa tenuiflora* hay, prepared from leaves and branches up to 8 mm in diameter from plants in full vegetative growth with an average height of 3.0 m. The material was chopped, air-dried on plastic tarps, turned every two hours, and covered overnight until reaching the hay stage. Once dried, it was processed through a forage mill with 5 mm and 2 mm sieves. Tannin extraction followed the method described by Chaves [25], based on Inô [16].

**Figure 1:**
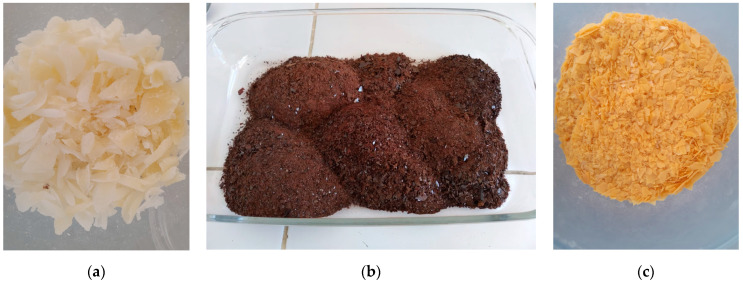
Materials used in the production of escape lysine; (**a**) carnauba wax; (**b**) dry tannin extracted from *Mimosa tenuiflora*; (**c**) escape lysine added with tannin as an adjuvant used in the sheep diet.

Microencapsulation of lysine as an escape protein source used carnauba wax as a shell and it was carried out following the fusion emulsification technique [4,11]. After obtaining the tannin extract, the CW (carnauba wax) and lysine (L-Lysine; Monohydrocloride; CJ Brazil, Piracicaba, Sao Paulo, Brazil) were weighed on an analytical balance at a 2:1 ratio (66.6% to 33.3%), and 3% tannin (based on the wax mass) was added according to Ino et al. [16], as described in Figure 1. The formulations were prepared using the emulsification technique, followed by oven drying, with the addition of 2.5% soy lecithin as an emulsifying agent [4,11]. To prepare the microencapsulated products, lysine and the corresponding tannin were weighed on an analytical balance, placed in beakers, dissolved in distilled water, and melted in a water bath at 65 °C and 85 °C, respectively.

The emulsions were prepared under heated conditions to prevent rapid solidification. The lysine solution was heated to match the temperature of the molten wax and then gradually added to the wax with constant stirring using a glass rod for 10 min. The material was subsequently dried in a forced air circulation oven at 55 °C for 6 h, yielding the microencapsulated product (escape lysine). The final product was crushed, stored in an appropriate container, and maintained at room temperature.

The experimental diets had a forage-to-concentrate ratio of 40:60, provided as a total mixed ration formulated to meet weight gain requirements according to NRC [1] recommendations, aiming for an average daily weight gain of 250 g. The forage component consisted of Tifton-85 hay (*Cynodon* spp.) and Buffel grass hay (*Cenchrus ciliaris* L.). The concentrate was composed of soybean meal, ground corn, and mineral mixture. Diets were offered twice daily at 08:00 and 15:00, with intake recorded daily to ensure 10% feed remnants. Water was provided ad libitum.

Samples of the hays, concentrate ingredients, and refusals were collected daily for intake adjustment and a weekly sample was composed for subsequent analysis. Then, samples were pre-dried in a forced air oven at 55 °C for 72 h, then ground in a Wiley knife mill to pass through a 1.0 mm sieve. Chemical composition analysis (Table 1) was conducted according to AOAC [26] methods, including dry matter (method 934.01), ash (method 942.05), crude protein (method 968.06), and ether extract (method 920.39).

Neutral detergent fiber (NDF) and acid detergent fiber (ADF) were determined following the method of Van Soest et al. [27], modified for non-woven fabric filters [28], with heat-stable amylase (Sigma A3306, Sigma-Aldrich, Steinheim, Germany) and expressed exclusive of residual ash (aNDF). Lignin was determined using AOAC [26] method 973.18, treating the acid detergent fiber (ADF) residue with 72% sulfuric acid. Non-fiber carbohydrates (NFCs) were calculated as per Mertens [29], incorporating NDF corrected for ash and protein into the formula.

Total digestible nutrient (TDN) contents were obtained from the following equation: TDN (g/kg) = (TDN intake/DM intake) × 100. The metabolizable energy (ME) was determined following the approach outlined by Weiss [30], utilizing the total digestible nutrient (TDN) content, considering 1.0 kg of TDN equates to 4409 kcal of digestible energy (DE), and ME was derived as ME = DE × 0.82.

### 2.3. Intake, Performance, and Ingestive Behavior

Nutrient intake was calculated as the difference between the total amount of each nutrient offered in the diet and the total amount remaining in the feed remnants, expressed in g/day. To determine the average weight gain of the animals, an initial weighing was performed, a partial weighing was performed every 15 days of the experimental diets, and a final weighing was performed at the end of the experimental period. The average daily gain (ADG) was obtained by dividing the total weight gain by the number of days the animals were confined. Feed conversion was calculated by DMI/ADG.

The animals’ ingestive behavior was assessed twice during the experimental period, on days 29 and 50. Observations were recorded at 10 min intervals over 24 h using the scan sampling method, as described by Martin and Bateson [31]. Data on the time spent eating, ruminating, or idling were collected by trained observers working in alternating shifts, positioned strategically to avoid influencing animal behavior. Artificial lighting was used for nighttime observations.

To estimate the average number of ruminal boluses per day, the number of chews per bolus, and the chewing time per bolus, evaluations were conducted during three distinct periods (08:00–12:00, 13:00–17:00, and 18:00–22:00) over a 24 h observation period. These assessments were performed using digital stopwatches.

The feeding and rumination efficiencies for dry matter (DM) and neutral detergent fiber (aNDF), expressed in kg/h, were calculated by dividing nutrient intake by the total time spent feeding and ruminating, respectively. The total chewing time was also determined. The ingestive behavior variables were calculated using the Poli et al. [32] recommendations: EE = DMI/ET (DM intake efficiency during feeding); RE = DMI/RUT (DM intake efficiency during rumination); TMT = ET + RT (total chewing time); EE (g DM/h) = eating efficiency; DMI (g DM/day) = dry matter intake; ET (h/day) = eating time; RE (g DM/h) = rumination efficiency; RT (h/day) = ruminating time; TCT (h/day) = total chewing time; Bolus amount (g DM/boli) = amount of ruminated bolus; and Bolus ruminated (n°/bolus) = number of chews per bolus (meristic mastication).

### 2.4. Dietary Selectivity

On days 30 and 37 of the experimental period, the feed troughs for each animal were weighed and sampled 12 and 24 h after feeding to analyze dry matter (DM) and particle size. A 250 g sample was collected for particle size assessment using the Penn State Particle Separator (PSPS), following the stratification method initially described by Lammers et al. [33] and later modified by Kononoff et al. [34]. This method employs a series of three sieves with mesh sizes of 19 mm, 8 mm, and 1.18 mm to separate the feed into four fractions based on particle size.

The physical effectiveness factor of fiber (pef) was calculated using two approaches: pef_8_, which includes the sum of the percentages of particles larger than 8 mm, and pef_1.18_, which accounts for the sum of particles larger than 1.18 mm. The physically effective neutral detergent fiber (peNDF) was determined by multiplying the NDF content of the sample by the corresponding pef value.

### 2.5. Physiological Variables of Sheep

Physiological variables were measured on the 15th and 35th days of the experiment, before and three hours after the animals were fed. These measurements were carried out during two time periods: hour 0 (from 7:00 to 10:00) and hour 3 (from 10:00 to 14:00). The respiratory rate was assessed by counting the number of respiratory movements over 30 s with the aid of a flexible stethoscope placed on the right side of the thoracic region. The number of movements was then multiplied by two to calculate the respiratory rate per minute.

**Figure 2:**
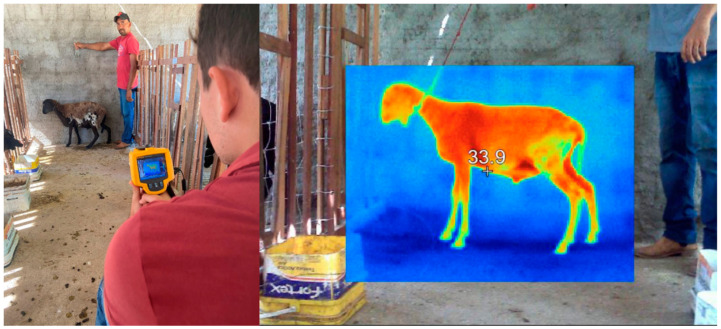
Surface temperature mensuration using an infrared thermographic camera.

Rectal temperature was recorded using a clinical veterinary thermometer with a range up to 44 °C. The thermometer was inserted into the animal’s rectum for two minutes, and the temperature was expressed in degrees Celsius (°C). Surface temperature (TS) was measured using an infrared thermographic camera (Fluke Ti 25 Series, Sao Paulo, Brazil), which has automatic calibration and an emissivity of 0.98, as recommended by the manufacturer for biological tissues (Figure 2). Thermograms were captured from both the right and left sides of the sheep and analyzed using Smartview software (version 4.3, Copyright© Fluke Corporation, Everett, WA, USA, 2006–2017). The thermographic variables, including maximum, minimum, and average surface temperatures, were obtained from the right and left flank regions of the sheep, as illustrated in Figure 2.

### 2.6. Digestibility Trial

For the digestibility trial, 24 sheep were allocated to metabolic cages for seven days (between days 40 and 47) for total collection of leftovers and feces. The diet was offered twice a day (at 8:00 am and 4:00 pm), with daily adjustments made to ensure that 10% of the previous day’s intake was left over.

Total samples of leftovers and feces were collected daily; then, at the end of the collection period, a composite sample was made for each animal, which was identified and frozen (only the feces), and then pre-prepared, dried in a forced air circulation oven at 55 °C, and ground in a Wiley-type knife mill (Tecnal, Piracicaba, São Paulo, Brazil) with a sieve of 1 mm in diameter, for further analysis. 

In order to determine the apparent digestibility coefficients (DCs), the following equation was used: DC = [(g of nutrient or analytical fraction ingested − g of nutrient or analytical fraction excreted in feces)]/(g of nutrient or analytical fraction ingested). Total digestible nutrients intake (TDNI) was obtained from the difference between the intake and that recovered in the feces of each nutrient, based on dry matter, according to the equation from Sniffen et al. [35]: TDNI (kg) = (digestible CP) + (2.25 × digestible EE) + (digestible NFC) + (digestible NDF).

### 2.7. Blood Metabolites

Blood samples from all sheep (*n* = 40) were collected and subsequently centrifugated in the morning at four hours after feeding on the first and last days of the experimental period following the methodology described by Melo et al. [36]. Serum biochemical measurements of total protein, albumin, urea, creatinine, aspartate aminotransferase (AST), alkaline phosphatase, gamma glutamyl transferase (GGT), calcium, phosphorus, and magnesium were performed using the colorimetric method, with commercial kits (Labtest, Bioclin, Sao Paulo, Brazil) using a Cobas C111 automated biochemical apparatus (Roche, Mannheim, Germany).

### 2.8. Environmental Variables

Throughout the experimental period, environmental data were continuously recorded using a HOBO^®®^ datalogger (model U12-013, Onset, São Paulo, Brazil). Two black globes were placed at animal height, one in a shaded area and the other in direct sunlight, to monitor conditions in both environments. The datalogger was set to log measurements every hour over a 24 h cycle. The Black Globe Temperature and Humidity Index (BGTHI) was calculated from the recorded data using the formula, according to methodology described by Buffington et al. [37]: BGTHI = [Tbg + (0.36 × Tdp) + 41.5]. Here, Tbg represents the black globe temperature, and Tdp is the dew point temperature.

### 2.9. Statistical Analysis

The data on intake, ingestive behavior, selectivity, serum metabolites, and physiological variables were analyzed using a completely randomized block design with the MIXED procedure (SAS Inst. Inc., Cary, NC, USA). The animal was considered the experimental unit for all variables. The statistical model used is described in Equation (1):

Y = μ + Bi + Dj + Eij(1)

where μ represents the overall mean, Bi is the block effect (i = 1–2), Dj is the diet effect (j = 1–5), and Eij is the residual error. The initial body weight (BW) was considered as a blocking factor for the distribution of experimental units. The block was included as a random effect.

Ingestive behavior data, serum metabolites, and physiological variables were analyzed as repeated measures using the MIXED procedure in SAS [38]. The statistical model for this analysis is described in Equation (2):

Y = μ + Bi + Dj + Sij + Tk + (DT)jk + Eijk(2)

where μ is the overall mean, Bi is the block effect (i = 1–2), Dj is the diet effect (j = 1–5), Sij is the residual error associated with the effect of the animal (block × diet), Tk is the effect of the observation/collection day (k = 1st and 2nd days), (DT)jk is the diet × observation day interaction, and Eijk is the residual error. The block was included as a random effect. Treatment means were compared using Tukey’s test, and significance was set at *p* ≤ 0.05.

## 3. Results

Regarding the nutrient intake, only for ether extract was a significant difference observed (Table 2). The ether extract intake was higher in the sheep fed escape lysine + tannin compared to the controls and free lysine group. The protein intake varied from 186 g/day to 202 g/day, and the neutral detergent fiber (NDF) ranged between 409 g/day and 435 g/day, showing minimal variation and resulting in statistical similarity among the treatments. There was no effect (*p* > 0.05) of the experimental diets on the animals’ performance variables.

No significant differences (*p* > 0.05) were observed in the feeding behavior variables, such as the time spent feeding, ruminating, and idling, total chewing time, DM and aNDF ruminating efficiency rates and chewing rates as the amount (g DM/bolus) and the bolus number (Table 3). However, the sheep fed with escape lysine presented a higher DM and aNDF eating efficiency (*p* ≤ 0.05) compared to those of the free lysine and control groups.

Twelve hours (12) after the morning feeding (Table 4), the diets significantly influenced the particle distribution (*p* ≤ 0.05), physical effectiveness factors, and the content of physically effective neutral detergent fiber (peNDF). The escape lysine and tannin blend diet (wax + lysine + tannin) resulted in lower percentages of particles retained on the 19 mm, 8 mm, and 1.18 mm sieves, with values of 11.92%, 13.82%, and 14.36%, respectively, though these differences were not significant compared to those of the control group. Approximately 60% of the basal diet particles exceeded those in the lysine-supplemented treatments but were comparable to the control (*p* > 0.05).

A similar trend was noted for effectiveness factors and peNDF content. At 24 h, the particle distribution was affected only with the 1.18 mm sieve. The escape lysine and tannin blend treatment showed a lower (*p* ≤ 0.05) particle percentage (14%) compared to the escape lysine without tannin (18%); no significant differences were observed to other treatments (*p* > 0.05). The peNDF selectivity was consistent across treatments.

No interaction (*p* > 0.05) between the treatments and evaluation time was detected for any physiological variable and, thus, the factors were evaluated separately. The rectal temperature was influenced by escape lysine supplementation (Table 5), with the animals receiving escape lysine showing the highest (*p* ≤ 0.05) temperature (39.1 °C), while those on free lysine had the lowest (38.9 °C). The other treatments yielded intermediate values.

Regarding time, the rectal and respiratory rates were significantly higher (*p* ≤ 0.05) three hours after sun exposure than at baseline (0 h or before feeding). The thermographic measurements of the animals’ lateral surfaces did not differ among the treatments, with a minimum temperature of 37.2 °C and a maximum of 38.3 °C (Table 6). Over time, the thermography results mirrored the respiratory and rectal temperature trends, with higher values (*p* ≤ 0.05) recorded at the 3 h mark: 39.8 °C and 38.6 °C for the left and right sides, respectively.

Dietary effects were observed on the crude protein (CP) digestibility, non-fibrous carbohydrate (NFC) digestibility, and total digestible nutrients (TDNs). The CP digestibility was greater in the escape lysine with added tannin and control group compared to the free lysine and escape lysine without tannin addition groups (*p* ≤ 0.05). The free lysine presented lower NFC digestibility (*p* ≤ 0.05) compared to that of the other treatments (*p* ≤ 0.05). In addition, the escape lysine with or without tannin presented a greater TDN digestibility compared to that of the control and free lysine treatments. And TDN digestibility was greater (*p* ≤ 0.05) in the escape lysine diet adjuvated with tannin compared to the escape lysine without tannin. No significant effects (*p* > 0.05) were found for the digestibility of DM, ash, organic matter, EE, NDF, or total carbohydrates (Table 7).

Serum concentrations of albumin (ALB), total protein (TP), urea (URE), triglycerides (TRI), aspartate aminotransferase (AST), gamma-glutamyl transferase (GGT), and minerals (phosphorus [P], calcium [Ca], and magnesium [Mg]) were not affected by the dietary treatments (*p* > 0.05) (Table 8).

## 4. Discussion

The ether extract intake was the only parameter significantly affected by the treatments, with a higher intake observed in the sheep fed escape lysine without (wax + lysine) or with tannin (wax + lysine + tannin), and the lowest EE intake recorded in those receiving free lysine. The increase in the EE intake by the sheep fed escape lysine compared to those fed free lysine occurred due to the use of carnauba wax and its impact on palatability and metabolism. Carnauba wax is composed mainly of fatty acids, fatty alcohols, and hydrocarbons that can impart a neutral or pleasant flavor to food [11,14], reducing rejection by sheep. In addition, its ability to encapsulate nutrients can mask undesirable flavors, such as those caused by tannins or other astringent compounds present in the diet [4,11]. Furthermore, carnauba wax, by encapsulating dietary ingredients, can protect lipids from oxidative processes or interactions with other compounds that could reduce their digestibility [39]. This increases the availability of ether extract in the small intestine, promoting greater EE intake.

The dry matter intake (DMI) and performance was similar among the experimental groups. The diet formulation followed NRC [1] recommendations, estimating an average DMI of 1050 g; however, the actual intake was greater in all of the dietary treatments, ranging from 1194 g to 1292 g. The inclusion of tannin extract did not promote an astringent effect in the sheep. This aligns with the findings by Da Silva Aguiar [17], who reported positive effects of incorporating *Mimosa tenuiflora* hay as a tannin source in sheep diets, achieving 1229 g of the DMI and 114 g of the crude protein (CP) intake at optimal tannin inclusion levels of 29.8 and 26.3 g/kg of the DMI, respectively.

In our previous research, Inô et al. [16] observed that the lysine encapsulation technologies demonstrated that incorporating tannin (3% of the encapsulated DMI) and carnauba wax effectively protected lysine from ruminal degradation, increasing the retention of dry matter and crude protein in microcapsules for intestinal absorption. These findings highlight the thermal stability of encapsulates during production and ruminal transit [4,16]. In our study, energy- and protein-balanced diets [40] supplemented with an escape lysine and tannin blend improved the CP digestibility compared to diets containing escape lysine without tannin or free lysine [41]. Additionally, the inclusion of escape lysine enhanced TDN utilization in the ruminal environment, regardless of tannin addition to the material, without affecting the temperature [42,43]. The best use and relationship (balance) of protein and energy in the diet of ruminants is very important because this supports microbial activity, optimizing volatile fatty acid production for efficient energy utilization [44], microbial protein availability [45], and escape lysine delivery to the abomasum [46].

The efficiency of carnauba wax encapsulation lies in its two primary components: fatty acid esters, which provide hardness, and hydrocarbons, which repel water and protect lysine from ruminal solubilization [4,16]. This protection enhances the proportion of retained dry matter and protein, ultimately improving the utilization of amino acids, such as lysine [2] and methionine [4,47], and supporting protein metabolism in ruminants.

The sheep feeding behavior correlated with the DM, OM, and aNDF intake and digestibility. The time spent eating, ruminating, and idling aligned proportionally with the intake, particularly with aNDF, which ranged from 407 to 435 g/day. The inclusion of the escape lysine improved the DM and aNDF eating efficiency. Araújo et al. [2] observed similar associations between the feeding behavior and dietary fiber levels in studies with encapsulated amino acids in sheep diets, consistent with Lima et al. [48], who evaluated diets containing various oils.

During the first 12 h post-feeding, the particle distribution highlighted the dietary effects on the CP and NFC digestion, emphasizing the importance of the energy–protein balance in particle selection. The sheep preferentially consumed concentrate-derived particles shortly after feeding, influenced by the diet composition. At 24 h, differences were observed only in the proportion of 1.8 mm particles. The escape lysine and tannin blend resulted in the lowest percentage (14.3%) compared to the escape lysine without tannin (18%). This highlights the effectiveness of material encapsulation, as diet selection was not influenced by the presence of tannin. In our study, tannin release into the ruminal environment did not lead to astringency, contrary to what might typically be expected.

In the rumen, tannins can bind to lysine, reducing its availability for absorption. This interaction underscores the importance of delivery systems, such as wax encapsulation, that protect lysine from tannin binding. It was anticipated that the main effects of both lysine and tannin, with this protective mechanism, would be on the digestibility and protein utilization efficiency, as the availability of escape amino acids in the small intestine would also support microbial protein production, helping sheep meet their protein requirements more effectively [49,50].

When formulating the diet, we aimed to determine whether the amounts of lysine and tannin used would influence palatability and feed selection. This effect was observed, as shown in the data in Table 4. The inclusion of tannin in the material promoted greater particle selectivity, even in small quantities, enhancing the material’s efficiency. The combined effects of tannins and lysine on the ingestive behavior may depend on the balance between the tannin concentration and lysine protection. However, properly formulated diets minimized the negative sensory impacts of tannins while ensuring adequate lysine bioavailability. The similar fiber content across the diets may also explain the lack of effect on the eating and rumination times [51]. Nevertheless, this did not preclude differences in the efficiency of the intake of dry matter (DM) or neutral detergent fiber (NDF).

The escape lysine diets showed greater CP and TDN digestibility compared to the free lysine and encapsulated lysine without tannin addition diets, which underscores the need for further research on amino acid supplementation in ruminants, particularly high-requirement sheep. The control diets outperformed the free lysine diet, likely due to lysine’s high ruminal solubility [52] and imbalances in the protein/lysine and energy availability for ruminal microorganisms. Nitrogen supplementation, whether protein-bound (e.g., lysine [53], methionine [54], or both [55]) or non-protein, such as urea [12,36,56], benefits from some form of protection to ensure intestinal absorption or slow ruminal release [11].

**Figure 3:**
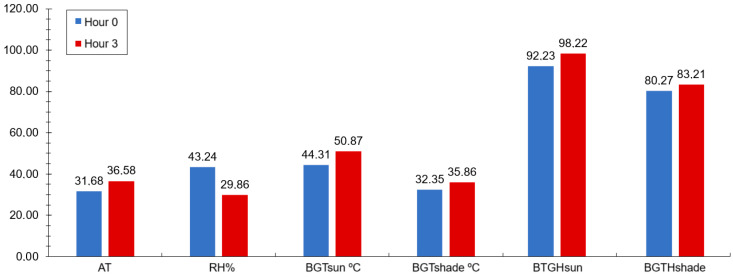
Ambient temperature (AT; °C), relative humidity (RH, %), black globe temperature in the sun (BGTsun; °C), black globe temperature in the shade (BGTshade; °C), black globe temperature-humidity index in the sun (BTGHsun), and in the shade (BTGHshade), measured before feeding (0 h) and three hours post-feeding in morning during experimental period (SEM = 5.89).

The observed increases in the rectal temperature, respiratory rate, and flank temperature three hours post-feeding in sheep can be attributed to the metabolic and physiological processes triggered by ruminal fermentation associated with the slight increase in climatic variables observed during the experimental period (Figure 3). After feeding, the availability of fermentable substrates, such as carbohydrates and proteins, increases in the rumen [57]. This leads to intensified microbial activity, which produces volatile fatty acids (VFAs) and gases as by-products [58]. The enhanced fermentation process elevates ruminal heat production, which is then reflected in the physiological parameters of the animals [59].

The increase in the rectal temperature is likely a systemic response to the metabolic heat generated also during ruminal fermentation [60]. This aligns with findings from previous studies indicating a time-dependent rise in body temperature following feeding in ruminants [60,61]. Similarly, the observed rise in the respiratory rate can be interpreted as a compensatory mechanism to dissipate the excess metabolic heat through evaporative cooling.

The elevated flank temperature, measured via thermography, further supports the notion of increased peripheral blood flow as part of the animal’s thermoregulatory response to mitigate the heat generated during digestion [57,60]. These changes are consistent with the diurnal feeding behavior of ruminants, where physiological parameters peak post-feeding due to the interplay between nutrient metabolism and heat dissipation mechanisms [61,62].

The isonitrogenous and isoenergetic nature of the diets was reflected in the similar biochemical parameters observed among the treatments, all within reference ranges. These results indicate adequate protein and energy metabolism and overall animal health [63]. Blood albumin, a key indicator of protein balance and nutritional status [64,65], ranged from 3.08 to 3.20 g/dL, consistent with reference values for sheep. The creatinine levels were also similar across the treatments (0.48–0.56 mg/mL), being below the reference range for sheep (1.2–1.9 mg/dL) [66]. While the nutrient intake typically influences creatinine levels, Liu and McMeniman [67] noted that healthy animals on balanced diets often maintain stable creatinine levels unless there are significant differences in muscle mass, which was not observed in this study.

Comparable values for urea, calcium, phosphorus, and magnesium across the treatments also align with the reference ranges, reinforcing the adequacy of the balanced diets in supporting renal and hepatic functions. These findings are consistent with previous studies [64,68], emphasizing the positive impact of energy–protein balance on excretory and protein metabolism.

As noted by Inô et al. [16], our study demonstrated the effectiveness of encapsulating escape protein to allow its passage to the intestine and delivering it efficiently for sheep nutrition. Supplementation with escape lysine microencapsulated in a lipid matrix of carnauba wax, regardless of the inclusion of tannin extract in the material, positively impacted the nutrient digestibility and eating efficiency. This was particularly evident when compared to diets containing free lysine. However, some aspects warrant further investigation to expand the understanding and applicability of these findings. Exploring the long-term effects of this supplementation on animal health, growth performance, and productivity metrics would provide valuable insights. While physiological parameters were evaluated, the only observed effect was a transient increase in the ruminal fermentation heat three hours post-feeding. Including additional parameters such as stress markers, hormonal profiles, and immune responses could offer a more holistic view of the supplementation’s systemic impacts. Another crucial aspect is the use of methodologies that enable the precise identification of how the intestinal release of escape lysine occurs, as well as the confirmation and quantification of the material utilized in intestinal passage. While digestibility studies provide valuable insights, it is essential to directly measure the amount of lysine being converted into muscle tissue and other metabolic activities for a more accurate understanding of its utilization.

It is also important to note that these results are specific to the dietary composition and experimental conditions used in this study. Variations in forage types, feed formulations, or management practices may influence outcomes. Future research could explore these variables to assess the broader applicability of the supplementation strategy. Such efforts would further enhance our understanding of the efficacy, practicality, and sustainability of using microencapsulated escape lysine and tannin extracts in diverse ruminant nutrition systems.

## 5. Conclusions

The supplementation of sheep diets with 1.34% of the total DM with escape lysine microencapsulated with carnauba wax, with the addition of tannin extract as an adjuvant, enhances the digestibility of crude protein and the TDN (energy), DM, and aNDF eating efficiency. This supplementation does not affect the animals’ ingestive behavior, sheep performance, or physiological parameters, except for the effect of feeding time, where the highest ruminal fermentation heat was observed three hours post-feeding. Therefore, the use of escape lysine in lamb diets should be tested at other doses and the cost should be considered for the use of confined lambs in finishing.

## 6. Patents

Two patents were filed resulting from the work reported in this manuscript. Registration number: BR10202301738, and registration number: BR102023017.

## Figures and Tables

**Table 1:** Proportion of ingredients and chemical composition of experimental diets.

Item	Experimental Treatments			
	Control	Lysine	^1^ Escape Lysine	^1^ Escape Lysine + Tannin
Ingredients				
Tifton-85 hay	20.0	20.0	20.0	20.0
Buffel grass hay	20.0	20.0	20.0	20.0
Ground corn	41.19	41.71	40.78	40.78
Soybean meal	17.5	16.5	16.5	16.5
Lysine	0.00	0.44	0.00	0.00
^1^ Escape lysine	0.00	0.00	1.34	0.00
^1^ Escape lysine + tannin	0.00	0.00	0.00	1.34
Mineral mixture	1.33	1.33	1.34	1.34
Chemical composition				
Dry matter	89.7	89.7	89.8	89.8
Ashes	6.75	6.70	6.70	6.70
Crude protein	13.3	13.3	13.2	13.2
Ether extract	2.42	2.42	2.38	2.38
aNeutral detergent fiber ^2^	51.99	51.62	51.32	51.32
Acid detergent fiber	24.4	24.3	24.2	24.2
Non-fiber carbohydrates	38.3	38.4	37.6	37.6
Total digestible nutrients	71.3	70.9	70.0	70.0
Metabolizable energy ^3^	2578	2564	2533	2533

^1^ Lysine protected in lipidic matrix of carnauba wax; ^2^ neutral detergent fiber assayed with a heat-stable amylase and expressed exclusive of residual ash; ^3^ Mcal/kg DM.

**Table 2:** Nutrient intake of sheep fed diets containing escape lysine protected in carnauba wax and tannin blend.

Variables	Control	Lysine	^1^ Escape Lysine	^1^ Escape Lysine + Tannin	SEM ^4^	*p*-Value ^5^
Intake (g/d)						
Dry matter	1292a	1194a	1241a	1272a	86.4	0.862
Organic matter	1220a	1127a	1172a	1202a	81.4	0.863
Ash	72.6a	66.1a	68.9a	70.0a	5.05	0.839
Crude protein	202a	186a	195a	198a	11.9	0.799
Ether extract	37.8bc	34.8c	47.5ab	48.7a	2.58	0.002
aNeutral detergent fiber ^2^	435a	407a	409a	419a	36.5	0.948
Non-fibrous carbohydrates	545a	499a	521a	536a	31.6	0.759
Total carbohydrates	980a	907a	930a	955a	67.0	0.880
Total digestible nutrients	892a	847a	869a	890a	87.4	0.292
Metabolizable energy ^3^	3.40a	3.00a	3.00a	3.11a	0.14	0.153
Performance						
Inicial body weight (kg)	23.8a	22.6a	23.3a	23.5a	1.19	0.903
Fial body weight (kg)	36.0a	34.4a	36.2a	35.1a	1.31	0.767
ADG (kg/d)	0.227a	0.220a	0.238a	0.216a	0.01	0.626
Feed conversion (g/g)	5.35a	5.26a	5.17a	5.58a	0.36	0.875

^1^ Lysine protected in lipidic matrix of carnauba wax; ^2^ neutral detergent fiber assayed with a heat-stable amylase and expressed exclusive of residual ash; ^3^ Mcal/day; ^4^ SEM = standard error of the mean; ^5^ means with different letters on the line indicate difference between treatments according to Tukey’s test (*p* ≤ 0.05).

**Table 3:** Ingestive behavior of sheep fed diets containing escape lysine protected in carnauba wax and tannin blend.

Ingestive Behavior	Control	Lysine	^1^ Escape Lysine	^1^ Escape Lysine + Tannin	SEM ^3^	*p*-Value ^4^
Behavioral events (min/day)						
Eating	197a	173a	159a	177a	17.39	0.494
Ruminating	559a	522a	534a	535a	19.29	0.590
Idling	684a	745a	747a	728a	25.19	0.273
Efficiency rates (g/h)						
DM eating	394c	414b	468a	431ab	9.84	0.002
DM ruminating	139a	137a	139a	143a	8.74	0.211
aNDF ^2^ eating	132c	141b	154a	142ab	7.99	0.027
aNDF ^2^ ruminating	46.7a	46.8a	46.0a	47.0a	3.88	0.225
Chewing rates						
Bolus amount (g DM/bolus)	3.70a	3.45a	3.87a	3.76a	0.31	0.578
Bolus ruminated (n°/d)	675a	624a	630a	651a	23.2	0.449
Total chewing time (min/d)	572a	584a	585a	581a	17.2	0.483

^1^ Lysine protected in lipidic matrix of carnauba wax; ^2^ neutral detergent fiber assayed with a heat-stable amylase and expressed exclusive of residual ash; ^3^ SEM = standard error of the mean; ^4^ means with different letters on the line indicate difference between treatments according to Tukey’s test (*p* ≤ 0.05).

**Table 4:** Particle size distribution, physical effectiveness factors (pefs), and physically effective neutral detergent fiber (peNDF) content of the diets containing escape lysine protected in carnauba wax and tannin blend.

Variables	Control	Lysine	^1^ Escape Lysine	^1^ Escape Lysine + Tannin	SEM ^2^	*p*-Value ^3^
Distribution of particles 12 h after feeding ^4^						
19 mm	22.64ab	28.72ab	33.30a	11.92b	5.70	0.040
8 mm	15.50ab	19.24a	16.03ab	13.82b	1.70	0.185
1.18 mm	16.27ab	19.31a	19.47a	14.36b	1.58	0.093
Bottom	45.61ab	32.73b	31.21b	59.90a	6.73	0.023
^5^ pef_8_	38.13ab	47.96a	49.33a	25.74b	6.38	0.059
^6^ pef_1.18_	54.40ab	67.27a	68.80a	40.10b	6.73	0.023
^7^ peNDF_8_	22.76ab	32.12a	32.47a	15.28b	4.93	0.066
^8^ peNDF_1.18_	32.34ab	45.07a	45.02a	23.54b	5.53	0.031
Distribution of particles 24 h after feeding						
19 mm	18.15a	30.46a	30.30a	15.62a	7.69	0.395
8 mm	10.90a	12.70a	12.93a	9.78a	1.16	0.204
1.8 mm	15.65ab	17.39ab	18.07a	14.30b	1.27	0.179
Bottom	55.31a	39.46a	38.71a	60.30a	7.86	0.152
pef_8_	29.05a	43.15a	43.22a	25.40a	7.74	0.256
pef_1.18_	44.69a	60.54a	61.29a	39.70a	7.86	0.152
peNDF_8_	17.10a	30.27a	28.91a	16.14a	6.27	0.261
peNDF_1.18_	26.33a	41.89a	40.64a	24.39a	6.81	0.173

^1^ Lysine protected in lipidic matrix of carnauba wax; ^2^ SEM = standard error of the mean; ^3^ means with different letters on the line indicate difference between treatments by Tukey’s test (*p* ≤ 0.05); ^4^ particle size distribution of the diet was measured using the Penn State Particle Separator [26]; ^5^ pef_8_ and ^6^ pef_1.18_ = physical efficiency factor determined as the proportion of particles retained on 2 sieves (8 and 19 mm) [25] and on 3 sieves (1.8, 8 and 19 mm) [26], respectively; ^7^ peNDF_8_ and ^8^ peNDF_1.18_ = effective neutral detergent fiber (NDF) of the sample multiplied by pef_8_ and pef_1.18_, respectively.

**Table 5:** Physiological and thermographic variables of sheep fed diets containing escape lysine protected in carnauba wax and tannin blend.

Variables	Control	Lysine	^1^ Escape Lysine	^1^ Escape Lysine + Tannin	SEM ^2^	*p*-Value ^3^
Physiological						
Respiratory rate (/min)	70.9a	66.0a	61.0a	63.4a	5.27	0.229
Rectal temperature (°C)	38.9ab	38.9b	39.1a	39.0ab	0.06	0.010
Thermography						
Left side temperature (°C)	37.2a	37.8a	38.3a	37.8a	0.05	0.062
Right side temperature (°C)	37.3a	37.8a	38.0a	37.7a	0.06	0.518

^1^ Lysine protected in lipidic matrix of carnauba wax; ^2^ SEM = standard error of the mean; ^3^ means with different letters on the line indicate difference between treatments according to Tukey’s test (*p* ≤ 0.05).

**Table 6:** Respiratory rate, rectal temperature, and lateral thermography in sheep as a function of feeding times.

Variables	0 h ^1^	3 h ^1^	SEM ^2^	*p*-Value ^3^
Physiological				
Respiratory rate (mr/min)	49.1b	81.6a	3.73	0.0001
Rectal temperature (°C)	38.8b	39.16a	0.04	0.0001
Thermography				
Left side temperature (°C)	36.7b	39.8a	0.03	0.0001
Right side temperature (°C)	36.8b	38.6a	0.04	0.0001

^1^ Time after morning feeding; ^2^ SEM = standard error of the mean; ^3^ means with different letters on the line indicate difference between treatments according to Tukey’s test (*p* ≤ 0.05).

**Table 7:** Digestibility coefficient (% of ingested) in sheep fed diets containing escape lysine protected in carnauba wax and tannin blend.

Digestibility (%)	Control	Lysine	^1^ Escape Lysine	^1^ Escape Lysine + Tannin	SEM ^3^	*p*-Value ^4^
Dry matter	63.2a	60.3a	64.2a	66.3a	2.23	0.6212
Ash	38.5a	34.8a	42.9a	48.2a	4.28	0.6331
Organic matter	64.7a	61.8a	65.5a	67.3a	2.17	0.8623
Crude protein	74.4a	64.4b	65.3b	72.4a	2.02	0.0354
Ether extract	63.2a	57.7a	65.5a	72.2a	3.91	0.1985
aNeutral detergent fiber ^2^	29.7a	35.5a	34.2a	35.4a	4.96	0.8974
Non-fibrous carbohydrates	88.7a	82.2b	88.0a	89.9a	1.50	0.0253
Total carbohydrates	62.7a	61.4a	64.9a	66.0a	2.22	0.8794
Total digestible nutrients	67.0c	68.0c	72.2b	76.0a	0.91	<0.001

^1^ Lysine protected in lipidic matrix of carnauba wax; ^2^ neutral detergent fiber assayed with a heat-stable amylase and expressed exclusive of residual ash; ^3^ SEM = standard error of the mean; ^4^ means with different letters on the line indicate difference between treatments according to Tukey’s test (*p* ≤ 0.05).

**Table 8:** Blood parameters of sheep fed diets containing escape lysine protected in carnauba wax and tannin blend.

Blood Metabolites	Control	Lysine	^1^ Escape Lysine	^1^ Escape Lysine + Tannin	SEM ^2^	*p*-Value ^3^
Albumin (g/dL)	3.20a	3.18a	3.08a	3.16a	0.007	0.693
Total protein (g/dL)	5.32a	5.46a	5.30a	5.44a	0.13	0.778
Urea (mg/dL)	35.9a	32.1a	33.2a	35.4a	1.67	0.344
Creatinine (mg/dL)	0.56a	0.54a	0.48a	0.51a	0.03	0.231
^4^ AP (U/L)	470a	574a	394a	566a	71.0	0.269
^4^ AST (mg/dL)	81.5a	78.8a	72.1a	73.6a	5.21	0.563
^4^ GGT (mg/dL)	45.7a	47.2a	48.9a	44.2a	3.77	0.850
Calcium (mg/dL)	9.60a	11.4a	10.9a	10.3a	0.78	0.394
Phosphorus (mg/dL)	7.81a	7.24a	7.12a	7.06a	0.35	0.419
Magnesium (mmol/L)	1.62a	1.48a	1.53a	1.75a	0.26	0.900

^1^ Lysine protected in lipidic matrix of carnauba wax; ^2^ SEM = standard error of the mean; ^3^ means with different letters on the line indicate difference between treatments accordingto Tukey’s test (*p* ≤ 0.05); ^4^ enzymatic activity = alkaline phosphatase (AP), aspartate aminotransferase (AST), gamma-glutamyl transferase (GGT).

## Data Availability

The data presented in this study are available upon request to the author for correspondence.
